# Extract of Cicuta in Gonorrhœa

**Published:** 1842-01

**Authors:** 


Dr. Staats, of Albany, has had such surprising success in the treatment of gonorrhœa, by the extract of cicuta, that he has addressed the Medical Society on the subject. In giving it to a patient with neuralgia, who happened also to have the other complaint, he was relieved of both by four grains every four hours till the system was under its influence. Since, he has cured several cases, giving the same dose once in four hours till dizziness was produced, to be followed by a dose of Epsom salts.—*Bost. Med. Jour.*

